# Integrin signaling in cancer: bidirectional mechanisms and therapeutic opportunities

**DOI:** 10.1186/s12964-023-01264-4

**Published:** 2023-09-28

**Authors:** Siyi Li, Chibuzo Sampson, Changhao Liu, Hai-long Piao, Hong-Xu Liu

**Affiliations:** 1 Department of Thoracic Surgery, Cancer Research Institute, Cancer Hospital of China Medical University, Liaoning Cancer Hospital & Institute, Shenyang, 110042 China; 2 Dalian Institute of Chemical Physics, Chinese Academy of Sciences, Dalian, 116023 China; 3 Department of Biochemistry & Molecular Biology, School of Life Sciences, China Medical University, Shenyang, 110122 China

**Keywords:** Integrin, Tumorigenesis, Bidirectional signaling mechanisms, Integrin-targeting drugs

## Abstract

Integrins are transmembrane receptors that possess distinct ligand-binding specificities in the extracellular domain and signaling properties in the cytoplasmic domain. While most integrins have a short cytoplasmic tail, integrin β4 has a long cytoplasmic tail that can indirectly interact with the actin cytoskeleton. Additionally, 'inside-out' signals can induce integrins to adopt a high-affinity extended conformation for their appropriate ligands. These properties enable integrins to transmit bidirectional cellular signals, making it a critical regulator of various biological processes.

Integrin expression and function are tightly linked to various aspects of tumor progression, including initiation, angiogenesis, cell motility, invasion, and metastasis. Certain integrins have been shown to drive tumorigenesis or amplify oncogenic signals by interacting with corresponding receptors, while others have marginal or even suppressive effects. Additionally, different α/β subtypes of integrins can exhibit opposite effects. Integrin-mediated signaling pathways including Ras- and Rho-GTPase, TGFβ, Hippo, Wnt, Notch, and sonic hedgehog (Shh) are involved in various stages of tumorigenesis. Therefore, understanding the complex regulatory mechanisms and molecular specificities of integrins are crucial to delaying cancer progression and suppressing tumorigenesis. Furthermore, the development of integrin-based therapeutics for cancer are of great importance.

This review provides an overview of integrin-dependent bidirectional signaling mechanisms in cancer that can either support or oppose tumorigenesis by interacting with various signaling pathways. Finally, we focus on the future opportunities for emergent therapeutics based on integrin agonists.

Video Abstract

**Supplementary Information:**

The online version contains supplementary material available at 10.1186/s12964-023-01264-4.

## Introduction

**Fig. 1:**
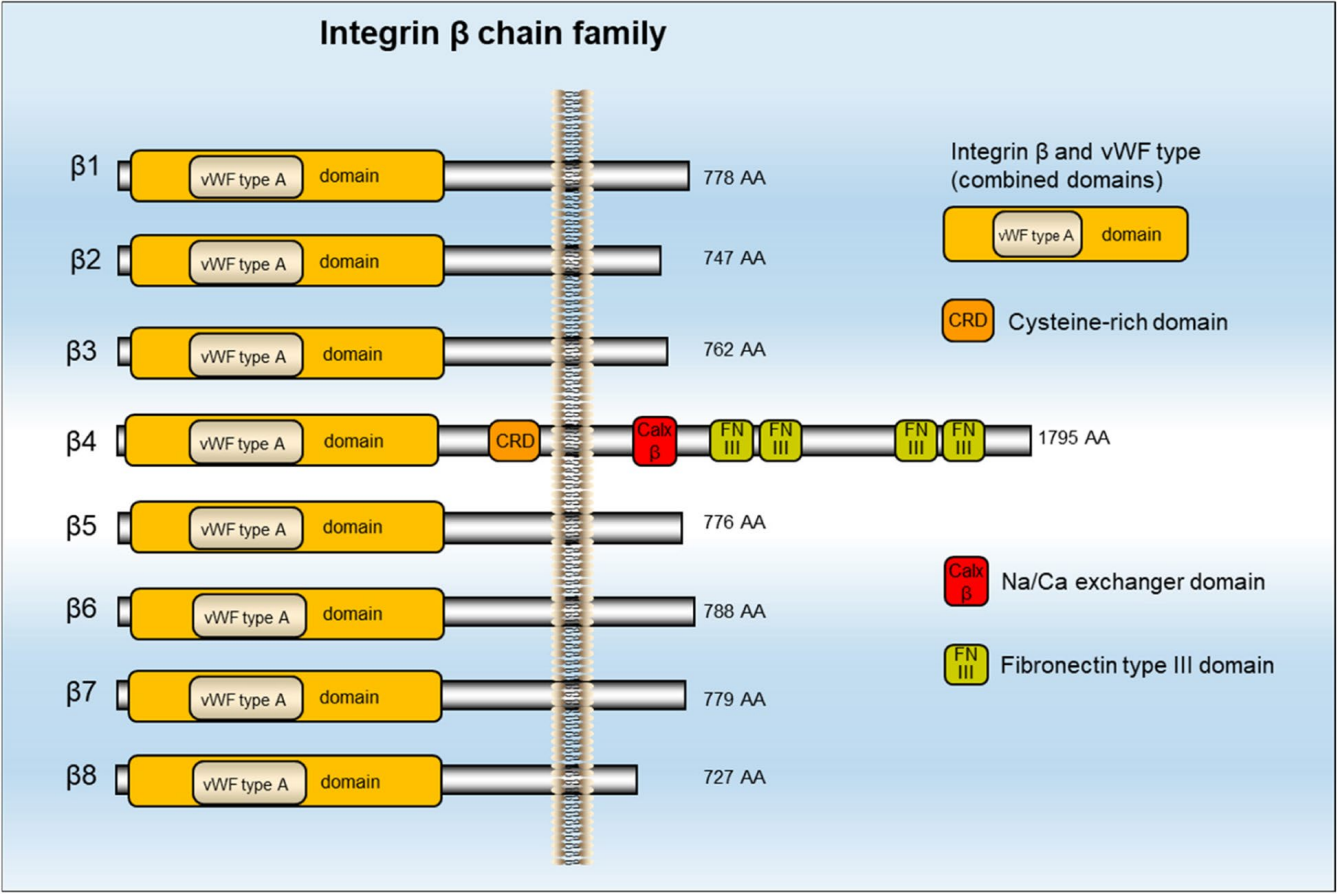
Structure of the integrin β domain

The first integrin is identified in 1986, as an integral membrane complex protein that plays a critical role in the association between the extracellular matrix (ECM) and the cytoskeleton [1, 2]. Integrins are a family of 24 heterodimeric receptors composed of stable covalently linked 18 α-subunits and 8 β-subunits, named according to their α/β-subunit compositions [3]. The α/β-subunits contain approximately 1000/750 amino acids, respectively [4]. Each subunit has a transmembrane helix and a cytoplasmic tail, which forms a ‘head’ (extracellular segment) supported by two α/β-subunit ‘legs’ (membrane-spanning regions and cytoplasmic tails) (Fig. 1). The head consists of Ig-like thigh and calf domains (C1 and C2)/Ig-like hybrid, EGF-like, and b-terminal domains (bTD), respectively [3–5]. Most integrins can mediate cell adhesion to various ECM proteins or ligands secreted by other cells and cellular counter receptors, such as intercellular adhesion molecule (ICAM) and vascular cell adhesion protein (VCAM) [6]. Correspondingly, many ECM and cell surface adhesion proteins can bind to a wide variety of integrin subtypes [4]. According to the characteristics of interaction, the contact between integrin and its ligand can be divided into four categories. Category I integrins, such as αV, α5β1, α8β1 and αIIbβ3, recognize vast ECM and soluble vascular ligands which contain RGD tripeptide active site [7, 8]. Category II integrins composed of α4β1, α4β7, α9β1, αEβ7 and β2, majorly identify fibronectin that have LDV peptide with RGD ligands related sequence [4]. Category III integrins comprising α1β1, α2β1, α10β1, and α11β1 are known to identify laminin or collagen that have collagenous GFOGER motif [9]; Category IV integrins like α3β1, α6β1, α7β1 and α6β4 recognize laminin, but the binding site is still unclear [4].

During the development process, integrin exerts an important role in cell rearrangement, migration and differentiation [10]. In myoblast and adipocyte differentiation, integrin α6 linking to laminin expression increases with a concurrent decrease in integrin α5 linking to fibronectin expression. However, when the dynamic balance is biased towards integrin α5, the cells may remain in the proliferation phase [11]. Integrin α6β4 induces epithelial cell proliferation and cooperation with its ligand laminin 5 regulates Ras-mediated keratinocytes differentiation in normal skin [12]. Gonadogenesis in C. elegans requires integrin (two α and one β subunit, αina-1/βpat-3)-mediated distal tip cell (DTC) migration. A dominant-negative form of integrin exchanges αina-1/βpat-3 and αpat-2/βpat-3 pairs cause DCT migration disorders. The integrin complementary expression is sufficient for the developmental process without ECM regulation [10, 11]. However, for muscle function, primordial germ cell (PGC) and neuronal cell migration, the regulation of ECM and surrounding cells is necessary for integrin-depended migration and development [11].

In addition, crosstalk between integrins and growth factor receptors (GFRs) is also necessary for the normal development process. For example, the phosphorylation of vascular endothelial growth factor receptor-2 (VEGFR-2) is regulated by integrin αvβ3 to recruit bone marrow cells in angiogenic sites [13]. Meanwhile, in human umbilical vein endothelial cells, VEGF2 activates integrin αvβ3 to regulate adhesion and migration [14]. Skin homeostasis needs the epidermal growth factor receptor (EGFR) association with integrin αvβ3 in human endothelial cells. Integrin-Src signaling-mediated adhesion to the basement membrane extracellular matrix leads to EGFR signaling activation by controlling four tyrosine phosphorylation [15]. Integrin αvβ6 and αvβ8 could activate TGFβ by promoting cleavage of latency-associated peptides (LAPs) to induce a conformational change [16, 17]. Consequently, the TGFβ up-regulates integrin α5β1 and αIIbβ3 in keratinocytes [18]. Of note, although GFRs and integrins co-localize at the cell membrane, they do not interact directly with each other, which needs to be explored further.

Integrins are an overarching regulator of pathophysiological progress, such as wound healing, tissue inflammation, tissue fibrosis, autoimmunity and metabolic disorders, in multicellular contexts of numerous diseases [19]. Integrin β1, as a subunit, comprises many heterodimers and plays a crucial role in wound healing due to its expression on various basement membrane cells and connective tissue cells [20]. Integrin αvβ1 can facilitate the differentiation of fibroblasts into myofibroblasts which may lead the wound closure, granulation tissue formation [21] and tissue fibrosis [22]. Integrin α5β1 connects with fibronectin to mediate keratinocyte proliferation which contributes to matrix adhesion during the invasion progress of connective tissue cells into the wound clot [23]. Integrin α9β1 is associated with wound re-epithelization [24] and integrin α11β1 is involved in the collagen remodeling of granulation tissue [25]. Integrin αv, the major regulator of TGFβ, associates with various β subunits for different roles. For instance, integrin αvβ1 induces ECM remodeling to regulate immune cell functions [26]. More so, integrin αvβ6 [27] and integrin αvβ8 [28] regulate immune response through activating TGFβ. Increasing persuasive research indicates that integrin αvβ3 is involved in inflammation response induced by macrophage activation, osteoclast development and inflammatory arthropathies [29–31]. Integrin αvβ3-mediated inflammatory process contributes to the pathogenesis of rheumatoid arthritis and the progress of related arthropathies [32]. Studies have shown that integrin regulates tissue fibrosis via binding to ECM to induce cell–cell and cell–matrix interactions [33]. Correspondingly, integrin αvβ5 [34], αvβ6 [35] and αvβ8 [36] mainly expressed in epithelial cells and fibroblasts, induce lung fibrosis through activating TGFβ. Moreover, integrin α1/5/6 [37], integrin β1 [38] and integrin β6 [39] are associated with fibrosis in the liver diseases such as chronic hepatitis B/C. Integrin β6 is also associated with primary sclerosing cholangitis (PSC) [39]. Integrin β6 [40], α3 [41] and α11β1 [42] are linked to human kidney fibrosis through inducing neovascularization and fibroblast differentiation. Given the contribution of integrin in multiple cell functions of both normal and diseased tissue, it is imminent to explore the involvement of integrin signaling in cancer growth and metastasis [1]. Here, we mainly review the newly discovered functions of integrin signaling in cancer and the emergent therapeutic opportunities dependent on mechanisms.

### Integrin signaling in cancer

#### Integrin signaling in cancer initiation and tumor growth

Dysfunction of normal cells acclimates the initiation and progression of malignancy [43]. Integrin binding with ECM is necessary for cancer initiating cells to sense and respond to the tumor microenvironment [44]. Studies indicate that integrins function as cell surface markers, as well as functional regulators of cancer stem cells, during cancer initiation [44]. Integrin α6 also called CD49f, a laminin-binding receptor, is the richest and most common cancer stem cell marker, expressed highly in many cancers including colorectal cancer [45], breast cancer [46], skin squamous cell carcinoma [47] and glioblastoma [48]. In glioblastoma stem-like cells (GSCs), integrin α6 regulates adverse stem-associated features according to the different molecular subtypes. Integrin α6 plays a crucial role in maintaining cancer stemness in proneural GSCs. However, in mesenchymal GSCs, integrin α6 does not have an impact on stemness and self-renewal. Silencing of integrin α6 affects DNA damage repair machinery and cell cycle thereby increasing mesenchymal GSCs radiosensitivity to ionizing radiation [49]. In triple-negative breast cancer (TNBC), integrin α6 high or low populations are isolated from TgMFT121, Brca1f/f p53f/f and TgWAP-Cre mice tumors by FACS and shown to activate focal adhesion kinase (FAK), but more significantly by high integrin α6 population cell. The activation of FAK induces the expression of Polycomb complex protein BMI, a stem cell factor, which contributes to the initiation of TNBC [50]. Notably, integrin β4, a combination of integrin α6, has been reported to be involved in lung development and normal lung stem cells. It has been used as a marker to isolate epithelial stem cells from the mouse lung tissue [51]. Indeed, studies have linked integrin β4 to self-renewal and proliferation of lung cancer stem cells during the lung cancer progression [43].

Integrins control most cell survival, proliferation and differentiation. They act through mediating cytoskeletal linkage between cell adhesion to ECM and nuclear envelope as well as mechanotransduction. Given its fundamental function, integrins play positive or negative roles in numerous diseases [52]. Different α/β integrin combinations mediate specificities in cancer [2, 53]. As type I transmembrane glycoproteins, the stable flux of integrins between plasma membrane and intracellular pools drive cells to bind to extracellular ligands and transmit intracellular signals [53]. The cooperation between integrins and receptor tyrosine kinases (RTKs) has been shown to drive intracellular signalings that promote cancer cell proliferation. For instance, integrin α6β4 amplifies oncogenic signaling via cooperating with EGFR, ErbB2 and Met RTKs [54]; integrin αvβ3 interacts with platelet derived growth factor (PDGF) receptor to enhance the growth of PDGF-oversecreting gliomas [55]. While the crosstalk between these integrins and RTKs amplifies the signaling output, other integrin subtypes work in parallel with RTKs-dependent ones to either promote or suppress tumorigenesis [1]. Integrin αvβ5, αvβ6, and αvβ8 activate TGFβ to induce growth inhibiting effect and mediate migration and invasion in tumor cells [1, 56]. However, since TGFβ itself plays a dual role in tumorigenesis and progression, and its function in tumor and stromal cells is not identical [52], the integrin and TGFβ crosstalk in tumor growth still needs to be explored deeply.

#### Integrin signaling in cancer adhesion, invasion and metastasis

**Fig. 2:**
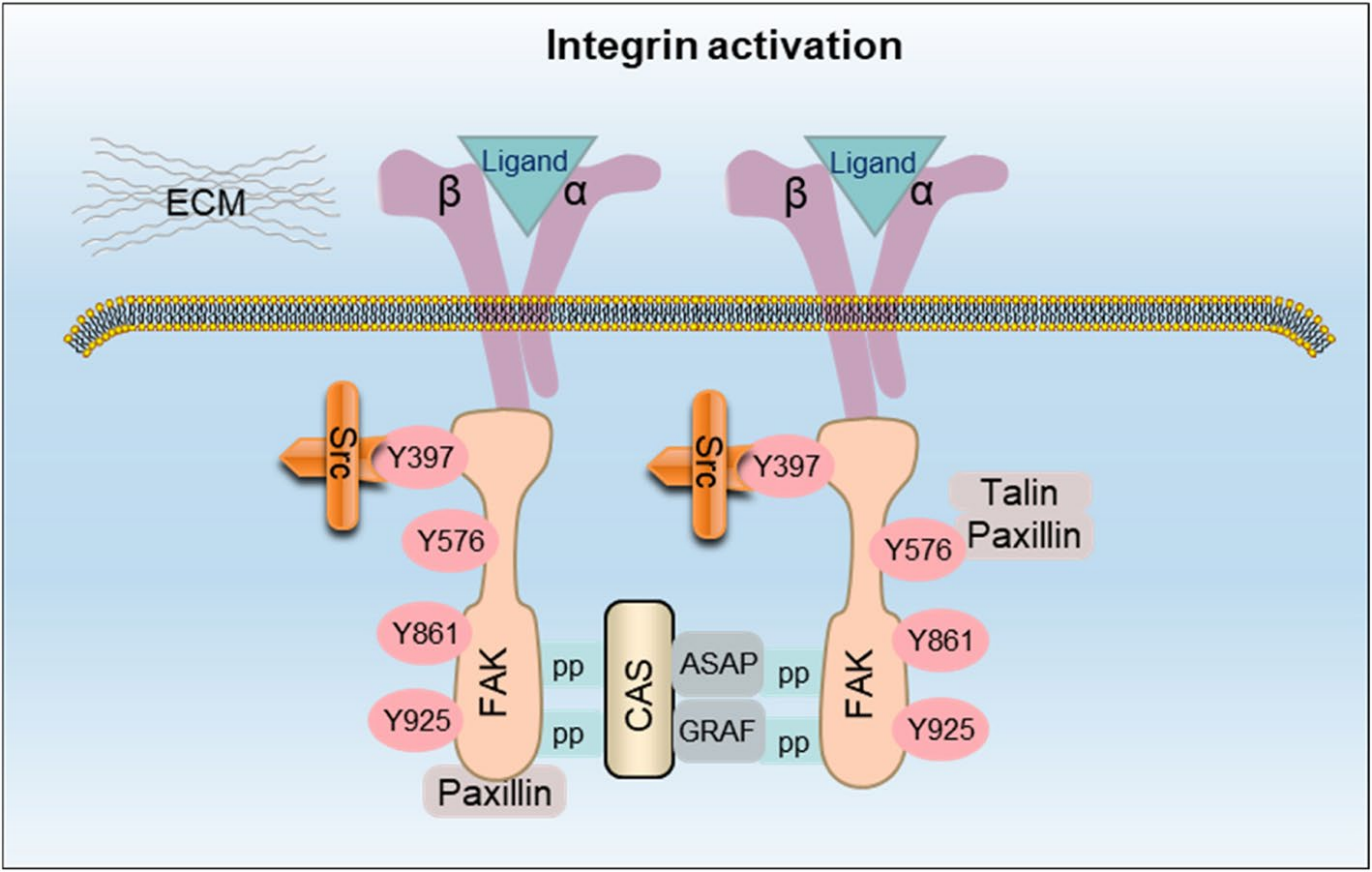
Integrin activation. Simplified binding interactions between integrin α/β subunit, extracellular ligand, FAK, Src and their activation components such as CAS, paxillin and talin

Increasing evidence indicates that integrin-mediated RTK signaling pathways are also implicated in tumor migration and invasion through cell–cell adhesion and cell motility. The activation of integrins were initiated through binding interactions involving integrin α/β subunits, extracellular ligands, FAK, Src, and associated activation components like Crk-associated substrate (CAS), paxillin, and talin (Fig. 2). Two important regulatory mechanisms induced by integrins have an imperative impact on this process. Initially, FAK signaling involved in RTKs induces tyrosine phosphorylation of E-cadherin-β-catenin complex. FAK acts as an integrin-regulated scaffold protein to recruit src-family kinases (SFKs) for focal adhesions which is crucial for cell invasion in cancers [12]. Additionally, integrin-linked kinase (ILK)-mediated epithelial-mesenchymal transition (EMT) exerts great contribution to increase cancer cell adhesion and invasion [57]. This process is majorly associated with Snail/Slug-reduced transcription and expression of E-cadherin [12] as well as AP1-induced matrix metalloproteinase 9 (MMP9) expression [57]. Integrin αvβ3 activates MMP2 specifically to facilitate cancer cell migration and invasion by leading the basement membrane degradation [58]. Simultaneously, this degradation develops new migration binding sites for other ones. The β4 tail of integrin α6β4 functions as an invasion signal adaptor and amplifier, promoting tumor invasion and inhibiting apoptosis. Therefore, the dysfunction of α6β4-RTKs signaling disrupts hemidesmosomes (adhesion complexes which regulate stable cell–matrix adhesion in the basement membrane by connecting intracellular keratin filaments with extracellular matrix) and leads to tumor invasion [12].

Besides, the engagement of integrin and ECM protein activates Rho- and Ras-GTPase which in turn controls cell adhesion, migration and invasion by regulating the dynamics of the actin cytoskeleton (outside-in signaling) [59]. Accordingly, both Rho and Ras superfamily proteins influence the interaction of integrin and ECM ligand (inside-out signaling) [60].

1. Outside-in signaling: This signaling mediates cellular responses induced by ligand binding to integrins. Integrin-induced adhesion is dependent on the relative activities of RhoA and Rac1. In the area of small nascent adhesions, Rac1 activation is accompanied by RhoA suppression. However, with large focal adhesion formation, RhoA but not Rac1 plays a major role. Activated integrin initiates two major downstream signaling pathways by activating FAK and Src kinases, gathered within the intracellular tails of β integrins. The FAK-Src complex in turn activates adhesion-associated adaptor proteins, such as paxillin and p130Cas subsequently binding and activation downstream adaptors [59]. What’s more, in the regulation of Rac-dependent adhesion by integrin engagement, ILK plays a circular role. ILK localizes to integrin β1 and β3 to form a scaffolding complex, which can activate PINCH1/2 and α/β parvin adaptor proteins [61].
2. Inside-out signaling: This signaling activates the ligand binding function of integrins. Rho- and Ras-GTPase encourage the binding of integrins to ligands by converting to a high affinity state. Ras, R-Ras and Rap1-GTPase may cause integrin activation which regulate cytoskeleton remodeling and integrin-dependent adhesion and migration on collagen [60]. Crosstalk and balance between Rho, Rac and Cdc42-GTPase also play a critical role in integrin-dependent invasion.

Cancer metastasis is the leading cause of cancer mortality. Metastasis is a cascading process including degrading the basement membrane and escaping from the primary tumor, accessing and surviving in the circulatory system, colonizing and proliferating in the parenchyma of the target organ [12, 43]. The properties of integrins suggest that they are crucial for the cascade process of cancer cell metastasis. Firstly, the interaction between integrins and ligands contributes to the degrading or remodeling of the ECM which is necessary for tumor cell escape [12]. Secondly, integrin binds various VEGFs and their receptors to form a complex which is required for active angiogenesis [62]. Finally, integrin-RTK signals regulate cell response to metastatic sites and initiate the metastatic cell survival, colonization and infinite proliferation in the targeting organ [63]. Notably, the metastatic process of several cancers presents extremely high levels of certain integrin forms. For example, hypoxia-inducibe factor (HIF) increases the expression of integrin α5β1 to accelerate metastasis of breast cancer towards lymph nodes and lung [64]. There is also research showing that integrin α5β1-mediated by kindlin-1 is involved in the early steps of breast cancer metastasis [65]. The crosstalk between integrin α4β1 and VCAM-1 induce melanoma and lymphoma metastasis to lung or spleen [66]. And as a pro-metastatic factor, L1-CAM interacts with integrin α5β1 and αVβ1 to promote numerous cancer metastasis, such as pancreatic ductal adenocarcinoma (PDAC), colorectal cancer as well as ovarian and endometrial cancers [67].

### Integrin-mediated downstream signaling pathways in cancer

**Fig. 3:**
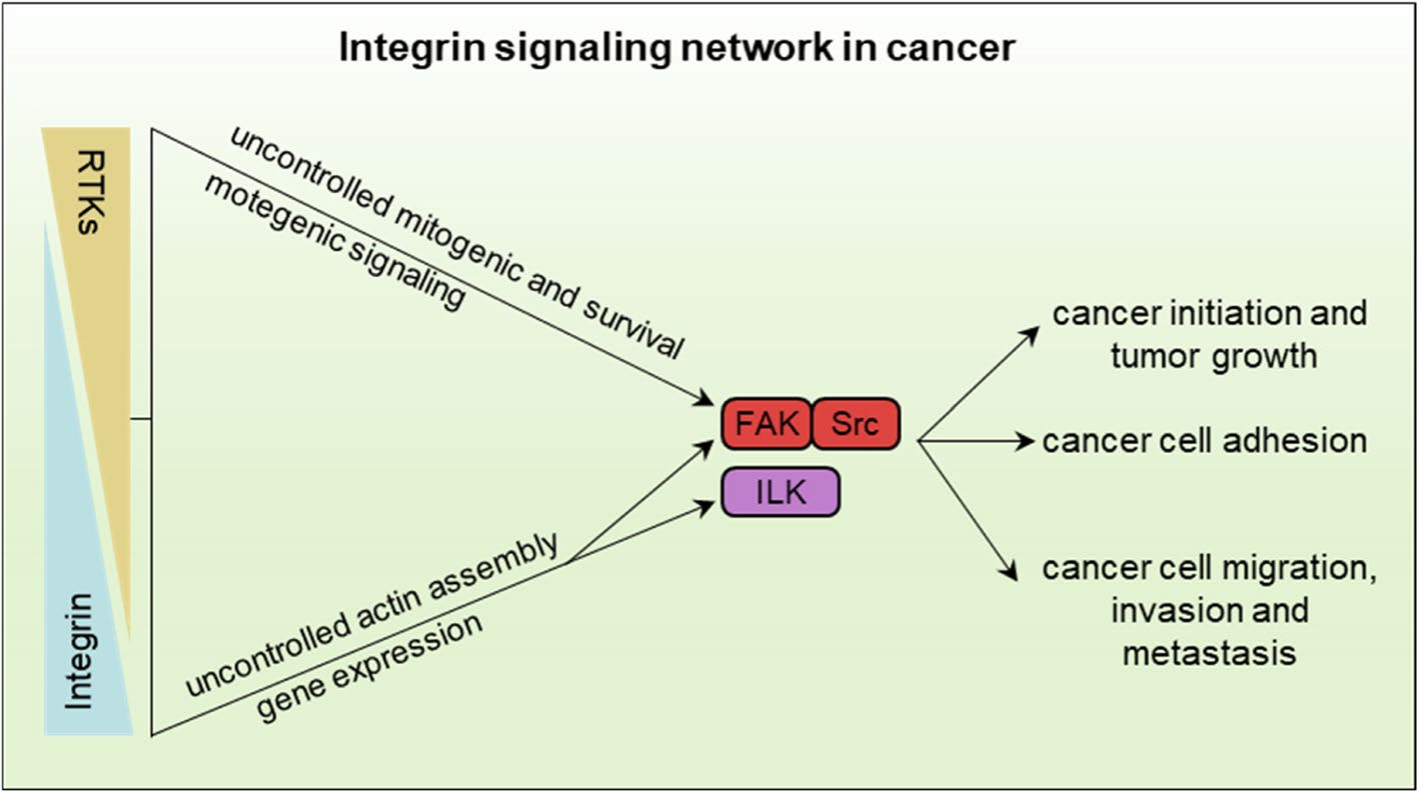
Integrin signaling network in cancer. Integrin interaction with RTKs mediates uncontrolled mitogenic and survival or uncontrolled actin assembly to regulate mitogenic signaling as well as related gene expression via FAK-Src (integrin/RTKs) or ILK (integrin) signaling. Activated integrin signaling controls cancer initiation and tumor growth, cancer cell adhesion and adhesion-induced migration, invasion and metastasis. It is associated with incidence and mortality of cancers

As a transmembrane receptor, integrin senses the content and stiffness of the surrounding ECM and biochemical signaling to mediate intracellular signal transduction, which occurs from adhesion sites or endosomes. The binding of ECM ligand (outside-in signaling) and specific cytoplasmic activators (inside-out signaling) to integrin lead to integrin-induced non-receptor tyrosine kinases activation. Studies show that the role of integrin in mechanotransduction and malignancy is bound to FAK-Src signaling activation [1]. FAK is the first kinase activated by integrin clustering via interaction between its C-terminal domain and integrin-containing components such as paxillin and talin. The activated FAK manifests as autophosphorylation at Y397, which leads to SFKs activation [68]. Well, the individual integrin subtypes including integrin β3 and integrin α4β1 directly stimulate the activation of Src by binding to its SH3 domain independent of FAK. Meanwhile, integrin α1β1, α5β1 and αvβ3 also activate the adaptor protein SHC through a palmitoylated SFK, such as Fyn or Yes. Numerous signalings, triggered by FAK-Src activation, such as Rho GTPase, TGFβ, Hippo, Wnt/β-Catenin and metabolism, require integrin-mediated ‘outside-in’ signals to drive diverse cellular functions [1]. As downstream pathway initiated by integrin, the FAK-Src or ILK signaling triggers uncontrolled mitogenic and survival or uncontrolled actin assembly to regulate mitogenic signaling as well as related gene expression, which subsequently regulates a series of cellular processes and biological events, including cell survival, proliferation, migration, self-renewal, EMT, and cell stemness maintenance [69] (Fig. 3).

#### Integrin-mediated Rho GTPase signaling

**Fig. 4:**
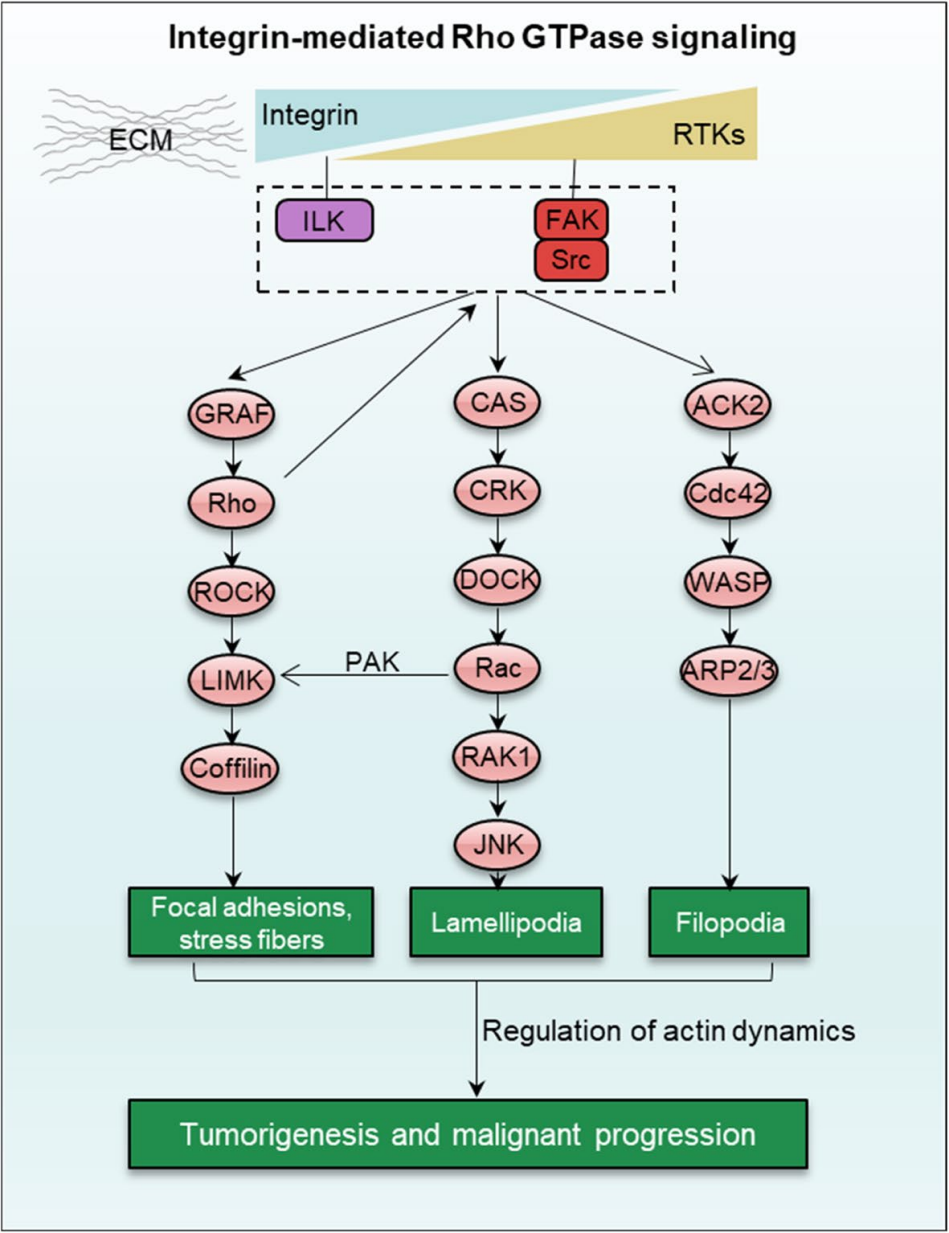
Integrin-mediated Rho GTPase signaling. Integrin associate with RTKs to mediate Rho GTPase subfamilies (RhoA, Rac and Cdc42) signaling via FAK-Src (integrin/RTKs) or ILK (integrin). Activation Rho GTPase signaling controls extension of lamellipodia and filopodia to form focal adhesions and actin stress fibers. Integrin association with the actin cytoskeleton by Rho GTPase is important for tumorigenesis and migration

Rho GTPase, a family of small G proteins, plays a crucial role in modulating cytoskeleton dynamics, associated with cell polarity, motility, growth, proliferation and survival. In human, 20 Rho GTPase subfamilies have been verified and Rho (RhoA/B/C), Rac (Rac1/2/3/G) and Cdc42 (Cdc42, RhoQ/J) are well-studied Rho GTPase [70]. Rho GTPase signaling is mainly activated by extracellular signals such as cytokines, ECM protein or mechanical signal such as integrin. The function of these Rho GTPase in cell contractility and polarization is tightly regulated by integrin-induced tyrosine phosphorylation of FAK [71]. Integrin triggers FAK-Src signaling and subsequently leads Rho, Rac and Cdc42 activation. The activated Rho GTPase controls extension of lamellipodia and filopodia to form focal adhesions and actin stress fibers, which induces tumorigenesis and maligant progression, such as cell migration and invasion in cancer (Fig. 4). In breast cancer cells, integrin regulates epithelial adhesion, polarity and invasion by inducing mechanotransduction and Rho activation [72]. Meanwhile, FAK-induced Rho signaling regulates cell proliferation and self-renewal which is associated with YAP/TAZ and AP-1 activation [73]. In cancer-associated fibroblasts (CAFs), integrin-induced Rho activation triggers CAFs to acquire a contractile phenotype to regulate tumor stromal remodeling, contributing to cell migration, invasion and metastasis [74]. In PDAC, integrin α6β1 regulates Rac1 and Rho activation to modulate cell migratory and TGFβ1 activation, respectively, via an Eps8/Abi1/Sos1 axis [75]. More so, integrin β1 concerts with Rac1 during peripheral regeneration to regulate fibroblast-derived TNC (tenascin-C)-mediated Schwann cell migration [76]. Notably, integrin-dependent cell adhesion and aggregation are also regulated by Rho GTPase. For instance, integrin αIIbβ3-induced platelet aggregation is suppressed by *botulinum* C3 exoenzyme, an inhibitor of Rho GTPase. This exoenzyme can also inhibit integrin αLβ3, αLβ2, α4β1 and α5β1, known to regulate lymphocyte and fibroblast activation [77]. Furthermore, FAK modulates Rho activation, in turn, this activation simultaneously increases cell contractility and disassembly of focal adhesion.

#### Integrin-mediated TGFβ signaling

Transforming growth factor beta (TGFβ) plays crucial homeostatic roles in the pathogenesis of inflammation and fibrosis [78]. There are three isomers of TGFβ (TGFβ1, TGFβ2 and TGFβ3) and high homologous and bind to the same TGFβ receptor (type I and type II). Growing evidence indicates that TGFβ spatiotemporal activation controlled by integrin has emerged as an important mechanism during a series of pathophysiological processes, including immunity, inflammation and fibrosis [19, 78]. Integrin αvβ6 is exclusively expressed in epithelial cells as a fibronectin receptor. Integrin αvβ6 perceives the surrounding signals and subsequently activates TGFβ in its LAPs. In normal epithelial cells, the crosstalk between integrin αvβ6 and TGFβ is critical for maintaining the tooth amelogenesis and periodontal health, hair follicle stem cell quiescence and suppression of intestinal epithelial cell inflammation to form epithelial barrier. On the other hand, in invasive cancers, the initiation of integrin αvβ6-mediated TGFβ signaling is associated with aggressive cancer and poor patient survival [79]. Other integrin-TGFβ complexes, besides integrin αvβ6, also exert a dual role that changes depending on the stage of cancer progression. In the early stage, integrin-TGFβ complex plays a cancer-suppressing role via activation of the anti-proliferative cytokines [79], while it acts a cancer promoter in the advanced stage of epithelial cancers [80].

**Fig. 5:**
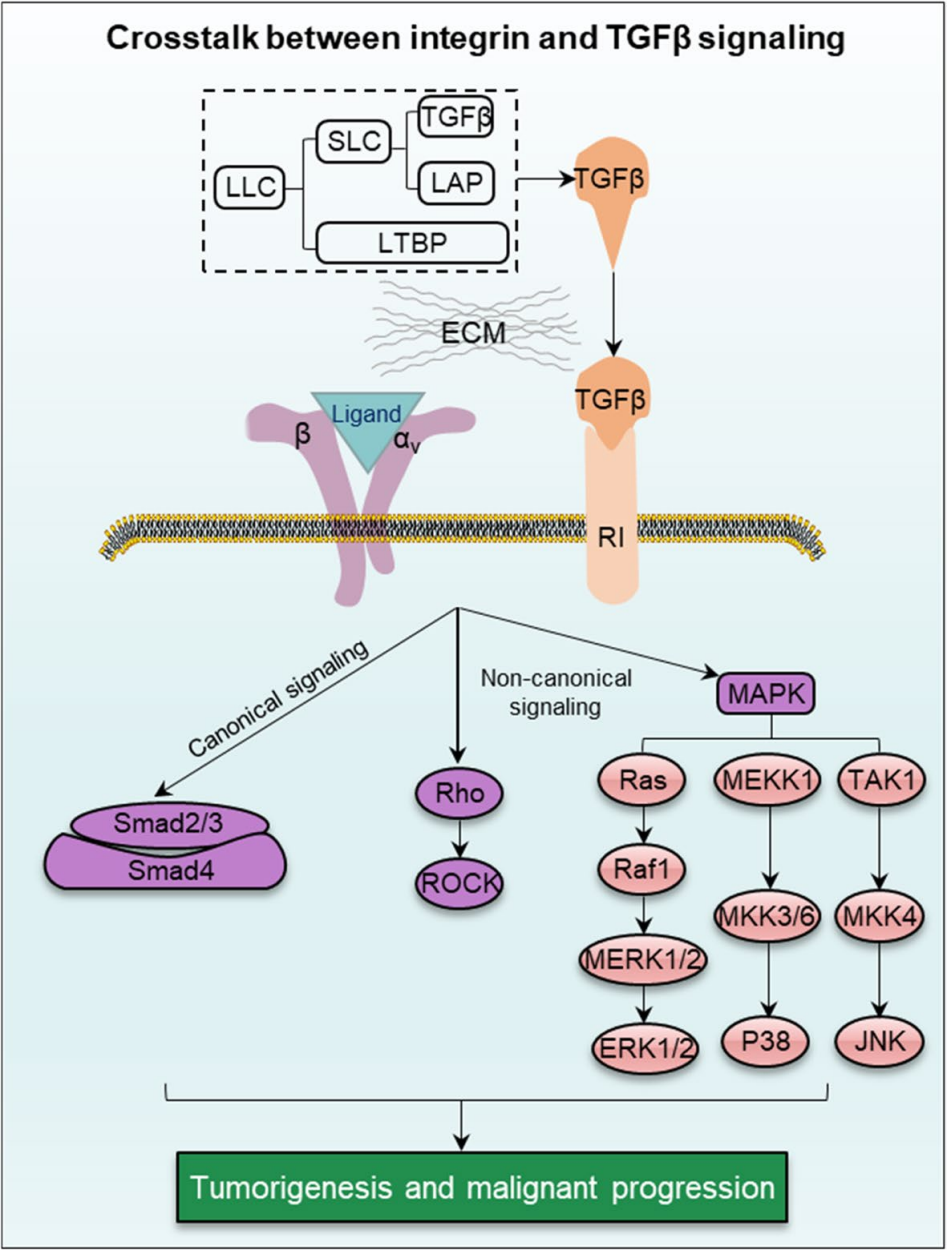
Crosstalk between integrin and TGFβ signaling. The combination of integrin and TGFβ signaling triggers canonical (Smads) and non-canonical (Rho GTPase and MAPKs) to induce EMT and malignant progression in cancer

During the wound healing of oral mucosa and skin epidermis, integrin αvβ6-TGFβ crosstalk is specifically increased. It is consistent with basement membrane regeneration, granulation formation and connective tissue remodeling [79]. During the development and genesis of fibrosis, integrin αvβ6-TGFβ complex drives fibrosis via Smad-dependent or non-Smad signaling, exerted by kinases and Rho GTPases [19]. These two different signaling pathways are crucial for the severity of fibrosis. However, not all TGFβ-mediated fibrosis is associated with integrin αvβ6. There is growing evidence that blocking integrin αvβ1, α3β1, αvβ3, αvβ5 and αvβ8 can be effective in the context of fibrosis. It works by controlling the differentiation of fibroblasts into myofibroblasts or the process of EMT in pulmonary fibrosis diseases [80]. In the immune system, integrin αvβ6 and αvβ8 are pivotal regulators to release TGFβ for either encouraging or inhibiting immune responses. Activated TGFβ triggers canonical (Smads) and non-canonical (Rho GTPase and MAPKs) signaling pathways to induce EMT and malignant progression in cancer (Fig. 5). Futhermore, modulation of activated TGFβ not only hampers the function of innate immune cells, but also exerts control over the recruitment, retention, and activation of immune cells, potentially culminating in the onset of severe autoimmunity [81].

#### Integrin-mediated mechanical cues: Hippo signaling and Wnt/β-catenin signaling

As a stiffness-sensor molecule, integrin not only acts as the physical scaffold between the extracellular stiffness and intracellular actin cytoskeleton, but also transduces the extracellular stimulation into cell to mediate mechanotransduction [82]. Several specific integrin subtypes are extremely involved in mechanical cues-mediated Hippo and Wnt/β-catenin signaling activation.

##### (1) Integrin-mediated Hippo signalling

**Fig. 6:**
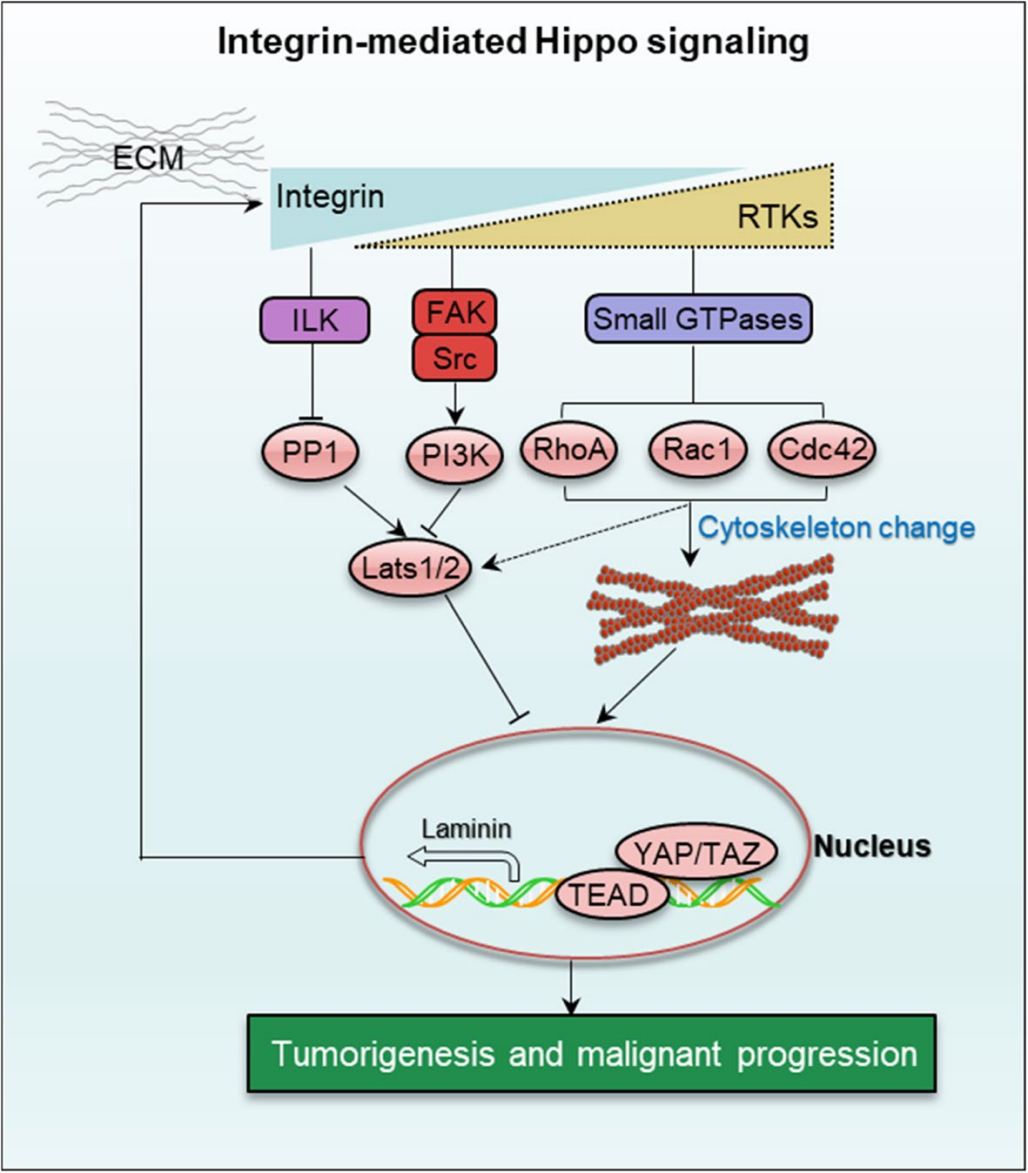
Integrin-mediated Hippo signaling. Integrin senses the extracellular mechanical cues to regulate the on/off switch of Hippo signaling. The Hippo core kinase cascade may be dispensable in the process of YAP/TAZ nuclear translocation by integrin activation. However, Rho GTPases, particularly RhoA contributes to YAP/TAZ dephosphorylation via regulating actin cytoskeleton dynamics

The Hippo signaling is first discovered in Drosophila. Due to the high conservation of the Hippo pathway in mammals, an analogous Hippo core kinase cascade exists which consists of Mst1 and Mst2 (Hpo homolog), WW45 or Sav1 (Sav homolog), Last1 and Lats2 (Wts homolog) and Mob1 (Mats homolog). The kinase cascade phosphorylates YAP (Yki homolog) and leads to YAP/TAZ co-activator inactivation and this inactivation inhibits its nuclear-cytoplasmic translocation [83]. Several reports have demonstrated that YAP/TAZ is an overarching regulator for stretching forces, epithelial sheet shape and surrounding ECM stiffness in multicellular environment. For instance, integrin β5 regulates ECM-mediated macrophage polarization via FAK-ERK1/2 pathway [84]. Integrin β1-Src complex interacts with the basal extracellular stiffness in skin cancer, and induces YAP/TAZ nuclear translocation in basal layer cells. Integrin β1 skin-conditional knockout phenotype is similar to YAP/TAZ skin-specific loss [85]. During the vascular remodeling, thrombospondin-1 (Thbs1) responds to the cyclic stretch and acts as a matrix sensor to induce YAP nuclear location via activating integrin αvβ1-mediated Rap2-dependent manner [86]. In Ewing sarcoma, EWS-FLI1-mediated tenascin-C promotes progression through integrin α5β1-induced YAP activation [87]. In gastric cancer, annexin A6 in extracellular vesicles from cancer-associated fibroblasts induces drug resistance through integrin β1-FAK-YAP signaling [88]. In atherosclerosis, integrin β3 directly senses the shear forces followed by activating YAP/TAZ-JNK cascade to mediate atheroprotective effect [89]. In the epidermal squamous cell carcinoma cancer stem cells, transglutaminase2 (TG2) controls ΔNp63α and interacts with integrin α6β4. The axis subsequently regulates FAK/Src and PI3K/PDK1 signaling which increases YAP and ΔNp63α feedback accumulation [47]. In colon cancer, ILK suppresses Hippo signaling by inhibiting its upstream molecule Merlin via regulation MYPT1-PP1 (a myosin light-chain phosphatase) [90]. In addition, Rho GTPases, particularly RhoA contributes to YAP/TAZ dephosphorylation via regulating actin cytoskeleton dynamics, Notably, this process might occur independently of the Hippo core kinase cascade (Fig. 6).

##### (2) Integrin-mediated Wnt/β-catenin signalling

**Fig. 7:**
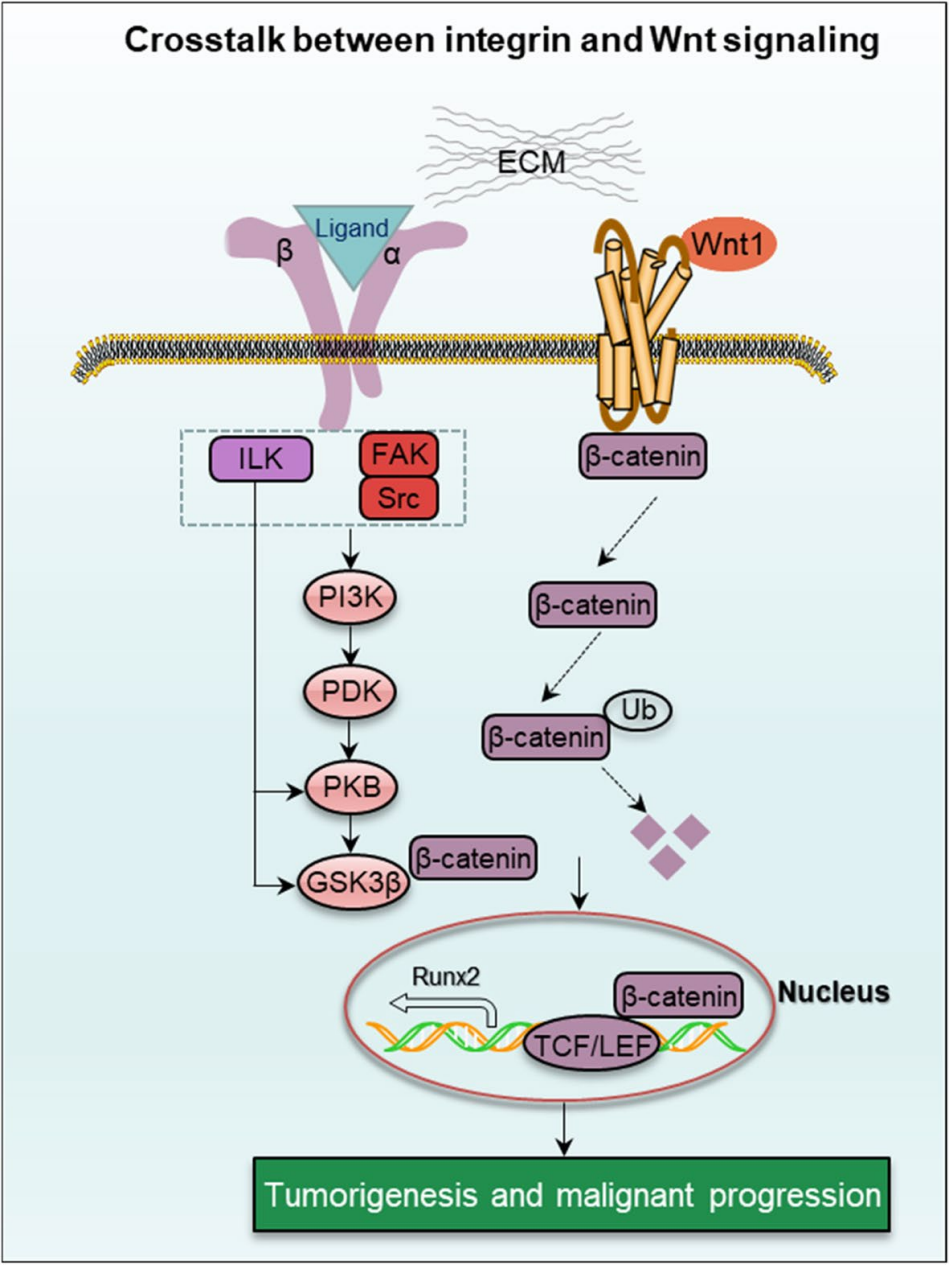
Crosstalk between integrin and Wnt signaling. The combination of integrin and Wnt signaling triggers PI3K activation further regulating GSK3β, Ub-induced β-catenin degradation, nuclear location and Wnt targeting gene expression

Wnt is an evolutionarily conserved signaling pathway involved in embryo development, tissue homeostasis and a variety of common diseases [91]. Wnt could be divided into non-canonical and canonical pathways. The canonical Wnt activation requires the cell membrane receptors binding to extracellular Wnt ligands, such as ECM, which immediately induces β-catenin nuclear translocation, known as Wnt/β-catenin signaling [92]. Integrin induces mechanotransduction through sensing shear stress, strain and ECM stiffness from extracellular cues. The extracellular mechanical cues play a crucial role in Wnt/β-catenin signaling activation during mechanosensing-mediate diseases [93]. The combination of integrin and Wnt signaling triggers phosphatidylinositol 3 kinase (PI3K) activation, subsequently this activations regulate glycogen synthase kinase 3β (GSK3β), ubiquitin (Ub)-induced β-catenin degradation, nuclear location and Wnt targeting gene expression (Fig. 7). Previous reports have indicated that integrin β1-dependent Wnt/β-catenin pathway participates in osteoblast differentiation, osteoblastogenesis and ostegenetic cell repair [93]. And this integrin β1/Wnt axis is also regarded as a potential therapeutic target in several cancers, such as colorectal and ovarian cancer [94, 95]. ECM-stimulating activated integrin β5 promotes hepatocellular carcinoma tumorigenesis through upregulating β-catenin level [96]. In TNBC, nanoparticles targeted integrin α5 inhibits β-catenin level to suppress tumor cell stemness and metastasis [97]. Notably, nanoparticles can also deliver integrin α9 to induce similar regulatory mechanism in TNBC [98]. ILK, as a mediator of cell–matrix interaction, is also associated with Wnt/β-catenin signaling in vascular smooth muscle cells as well as endothelial cell-related diseases [99].

#### Integrin-mediated metabolism in cancer

**Fig. 8:**
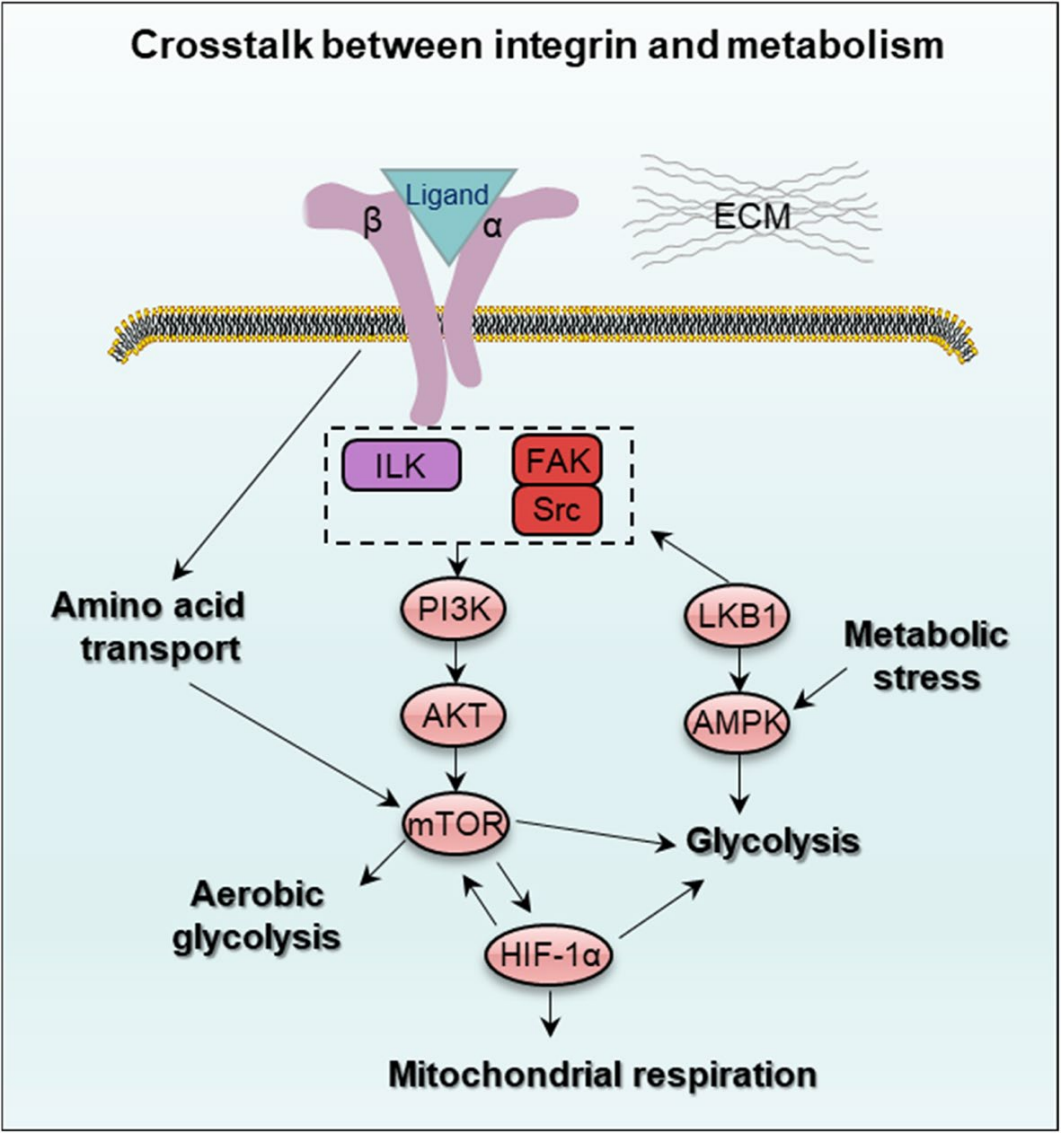
Crosstalk between integrin and metabolism including amino acid transport, aerobic glycolysis, glycolysis, glycolysis and mitochondrial respiration

Cellular metabolism under both physiological and malignant condition is extremely associated with extracellular matrix remodeling and integrin traffic, especially in epithelial and endothelial cells with fibroblasts [100, 101]. Cancer cells directly acquire nutrients from surrounding microenvironment via integrin-induced endocytosis of ECM [102]. Furthermore, cancer cells secrete exosomes to regulate normal cell metabolism and angiogenesis, which is important in biological functions of immune and cancer cells. The metabolic crosstalk, in turn, induces ECM remodeling and vascular barrier impairment [53]. Integrin regulates mTOR signaling by activating PI3K/AKT or taking part of amino acid transport. mTOR activation by integrin directly regulates aerobic glycolysis, glycolysis or influences HIF-1α to induce mitochondrial respiration. Besides, HIF-1α, metabolic stress or the AMPK-activating kinase (LKB1) also increase AMPK activity to promote glycolysis and regulate FAK activation (Fig. 8). In TNBC, integrin β4 is in a persistently high expression state and this mediates the metabolic reprogramming of CAFs. The integrin β4, via exosomes, initiates BNIP3L-dependent mitophagy and lactic acid accumulation-induced glucose metabolism [103]. In prostate cancer, exosomes transmitted by integrin αvβ3 [104] and integrin αvβ6 [105] arouse cancer cell aggression. In neutrophil-mediated inflammatory response, integrin β2 participates in the adhesion process between exosomes from neutrophils and endothelium [106]. There is also a report indicating that in neutrophil, integrin β2-dependent adhesion modulates cell energy metabolism through a small GTPase Arf6-induced pathway [107]. Integrin α4β1 is considered as a biomarker of the malignant transformation during the hematopoietic stem cell-renewal and differentiation [108]. During the atherosclerosis and cancer in small intestine, integrin β7 expressed in the gut intraepithelial T cells controls inflammation and metabolism [109]. In cervical cancer, high expression of PDL1 promotes glycolysis and lymph node metastasis through integrin β4-SNAL1/SIRT3 signaling pathway [110]. Chemoresistance, such as cisplatin resistance, is exaggerated when integrin β4 binds to paxillin (PXN) and FAK to form a focal adhesion complex in lung cancer. This complex increases Bcl-2 expression and dynamin-related protein 1 (DRP1) to modulate cisplatin resistance [111].

### Potential therapeutic target of integrins

In clinic, integrin is considered as a double-edged sword. On the one hand, integrin signaling activation promotes the resistance of chemotherapy and radiotherapy. However, since integrin is transmembrane protein sensitive to pharmacological treatment, disease therapeutic strategies targeting integrin may achieve considerable clinical success. Three classes of drug can target integrin: monoclonal antibodies, peptides and small molecule inhibitors [112]. To date, only few integrin targeted drugs including abciximab, eptifibatide, tirofiban, natalizumab, vedolizumab, lifitegrast (SAR-1118) and carotegrast (AJM-300) have been successfully marketed. These drugs mainly target αIIbβ3, αVβ3, α4β1, α4β7 and αLβ2 [113]. Recently, a comprehensive overview of integrin-targeting drugs undergoing clinical trials is presented in Table 1. Among these candidates, roughly twenty-five percent are currently in Phase I trials, while approximately sixteen percent have progressed to Phase III. Concurrently, some of the drugs are in the process of patient recruitment, while others have encountered challenges in advancing through the trial stages. Compared with monoclonal antibodies and peptides, small molecule inhibitors constitute the predominant portion of the ongoing clinical trials, due to their cost-effectiveness, safety profile, pharmacokinetic characteristics, and route of administration.

**Table 1:** Recent integrin-targeting drugs in clinical studies

Drug class	Drugs	Integrin targets	Indication	Delivery route	Study status	References
**Monoclonal antibodies**	SAN-300	α 1β 1	RA	IV	Phase I	[114]
	Vatelizumab	α2β1	MS and IBD	IV	Phase I	[115]
	OS2966	β1	Glioma	Intratumoural infusion	Phase I	[116]
	BG00011(STX-100)	αvβ6	IPF	SC	Phase II/I	[78]
	Volociximab	α5β1	NSCLC and AMD	IV	Phase II	[117, 118]
	Etaracizumab	αvβ3	Melanoma	SC	Phase II	[119]
	Intetumumab	Pan-αv	Melanoma and prostate cancer	IV	Phase II	[120, 121]
	Abituzumab	^Pan−αv^	Colorectal cancer and melanoma	IV	Phase II	[122, 123]
	Abrilumab	α4β7	UC	SC	Phase II	[124, 125]
	VPI-2690B	αvβ3	Diabetic nephropathy	SC	Phase II	(http://www.vascularpharma.com/science/vpi-2690b)
	ASP-5094	α 9β 1	RA	IV	Phase II	[126]
	Natalizumab biosimilar	α4β1, α4β7	IBD	IV	Phase III	[127, 128]
	Etrolizumab	α4β7, αEβ7	UC and Crohn’s	SC	Phase III	[129, 130]
**Peptides**	AXT-107	αvβ3, α5β1	DME, nAMD	Intravitreal Injection	Recruiting	[131]
	PN-943	α4β7	UC	Oral	Recruiting	[129]
	PTG-100	α4β7	UC	Oral	Phase II	[132]
	ATN-161	α5β1	Cancer, Crohn’s and SARS-CoV-2	IV	Phase II ?	[133, 134]
	Cilengitide	αvβ3, αvβ5	Glioblastoma	IV	Phase III	[135]
**Small molecules** **inhibitors**	MK-0429	Pan−αv	MBD	Oral	Not mentioned	[136]
	ELND-002	α4	MS	SC	Phase I	(https://adisinsight.springer.com/trials/700056998)
	7HP-349	αLβ2, α4β1	Solid tumor	Oral	Phase I	[137]
	GLPG-0187	Pan-αv, α5β1	Solid tumor	IV/Oral/SC	Phase I	[138]
	GSK3008348	αvβ6	IPF	Inhalation	Phase I	[139]
	THR-687	Pan-αv, α5β1	DME	Intravitreal injections	Phase I	(https://www.oxurion.com/content/oxurion-nv-expert-presentation-positive-topline-data-phase-1-study-evaluating-thr-687)
	Firategrast	α4β1	MS and Crohn’s	Oral	Phase II	[140]
	AXR-159	α4	DED	Topical	Phase II	(https://clinicaltrials.gov/ct2/show/NCT03598699?cond=AXR-159&draw=2&rank=1)
	RO-0506997	α4	MS	Oral	Phase II	(https://adisinsight.springer.com/trials/700201021)
	PLN-74809	αvβ1, αvβ6	IFP	Oral	Phase II	[141]
	OT-166	αvβ3, αvβ6, αvβ8	AMD	Topical	Phase II	(https://www.businesswire.com/news/home/20171218005625/en/SciFluor-Announces-Positive-Top-Line-Results-Phase-12)
	Risuteganib	αvβ3, αvβ5, α5β1	DME and dry agerelated macular degeneration	Intravitreal injections	Phase II	[142]
	SAR-1118	αLβ2	DED and conjunctivitis	Topical	Phase II	[143]
	MDL-819767	α4β1	Arthritis	Inhalation	Phase II	[144]
	TRK-170	α4β7	Crohn’s	Oral	Phase II	(https://clinicaltrials.gov/ct2/show/NCT01345799)
	RO-27–0608	α4	Asthma	Oral	Phase II	[145]
	Zalunfiban	αIIbβ3	STEMI with PCI	SC	Phase III	[146]
	AJM-300	α4	IBD, UC and Crohn’s	Oral	Phase III	[147]
	Orbofiban	αIIbβ3	Thrombosis	Oral	Phase III	[148]

*IV* Intravenous, *SC* Subcutaneous, *RA* Rheumatoid arthritis, *MS* Multiple sclerosis, *IBD* Inflammatory bowel disease, *IPF* Idiopathic pulmonary fibrosis, *NSCLC* Non-small-cell lung cancer, *AMD* Age-related macular degeneration, *UC* Ulcerative colitis, *DME* Diabetic macular edema, *nAMD* Neovascular AMD, *MBD* Metastatic bone disease, *DED* Dry eye disease, *STEMI* ST-elevation myocardial infarction, *PCI* Percutaneous coronary intervention

Targeting integrins beyond the ligand binding site (specifically the allosteric site), holds the potential to hinder integrin activation by either obstructing the orthosteric site or by maintaining the conformation in a low-affinity state. Within this context, monoclonal antibodies have been categorized into three distinct groups: inhibitory antibodies, activation-specific antibodies, and non-functional antibodies. The primary function of monoclonal antibodies lies in their capacity as competitive inhibitors, with a significant portion exerting allosteric inhibitory effects. These antibodies exhibit specificity for discrete regions within the integrin ectodomain, selectively targeting specific subunits or conformations.

Integrin αIIbβ3 is specifically expressed in platelets and megakaryocytes, aggravating cardiovascular and autoimmune diseases. Integrin αIIbβ3 antagonist, Abciximab, is the first drug used in thrombosis-associated disease. Due to the remarkable efficacy of abciximab, eptifibatide and tirofiban are developed immediately afterward [149, 150]. They inhibit platelet aggregation via binding to fibrinogen and other ligands that induce integrin αIIbβ3 activation in angiogenesis related disease [112, 150]. Talin, kindlin and other relatively uncommon proteins, such as ILK, β3 endonexin and vinculin are involved in integrin αIIbβ3 activation [151]. While other proteins including calcium and integrin binding protein 1 (CIB1) [152], docking protein 1 (Dok1) [153] and filamin [154] mediate integrin αIIbβ3 inactivation via binging either integrin αIIb or integrin β3 tail in the cytoplasmic region.

Integrin αVβ3 is mainly expressed in vascular smooth muscle cells and macrophages. Integrin αVβ3 mediates cell-dependent inflammatory angiogenesis, which is essential for the pathology rheumatoid arthritis and related arthropathies. Emerging evidence suggests that as an RGD-binding subfamily, integrin αVβ3 is also associated with ophthalmology and osteoporosis [150]. Vitaxin, a monoclonal antibody used in treatment of rheumatoid arthritis, antagonizes integrin αVβ3 by binding both integrin αV and integrin β3 tails. This disrupts the crosstalk of integrin αVβ3 and osteopontin and vitronectin. Integrin αV and β3 alone cannot be recognized by vitaxin [155].

Integrin α4β1, α4β7 and αLβ2 are leukocyte-specific protein, which contribute to immune response in inflammatory bowel disease (IBD), multiple sclerosis (MS) and dry eye disease (DED). Integrin α4β1 binding to VCAM-1 controls leukocyte diapedesis [156]. In addition, a fraction of integrin α4β1, expressed in endothelium, is also required to induce immune cell adhesion via VACM1, which plays a potential role in the infiltration of immune cells into central nervous system [150]. Integrin α4β7 is a leukocyte gut-homing receptor that binds mucosal vascular address in CAM1 to induce T-cell homing [157]. Integrin αLβ2 and α4β1 modulate T cell activation and adhesion to facilitate T cells infiltration into tumor cells [150]. Natalizumab is an extensive integrin α4 subtype inhibitor used in MS [158] and Crohn’s disease [159]. However, its limitation, including tendency to promote progressive multifocal leukoencephalopathy, and poor drug delivery system, makes it necessary to develop the gentler and more effective drugs [150]. Currently, a specific integrin α4β7 inhibitor, vedolizumab delivered subcutaneously has already been approved for clinical use in IBD, such as Crohn’s disease [160]. Meanwhile, drugs targeting integrin β7, like etrolizumab, is already in clinical study and is only effective against integrin β7, which has no effect on integrin α4β7. This specific feature likely provides unexpected benefits [157]. Another integrin antagonist, lifitegrast, prevents lymphocyte adhesion resulting in hindering T cell activation, releasing inflammatory factors and subsequently reducing T cell-induced inflammation by blocking the interaction of integrin αLβ2 and ICAM-1 in DED [161].

Integrin-ECM interaction triggers cell adhesion-induced drug resistance to chemotherapy, radiotherapy and targeted therapy. This unexpected drug resistance is due to the change of drug targets, substitutability of anti-apoptotic events and invalidation of cell death, especially in the treatment of cancer [43]. In breast cancer, integrin α6 mediates tamoxifen resistance via integrin α6/Src/AKT signaling [162]. Integrin β1 participates in lapatinib and transtuzumab resistance by activating human epidermal growth factor receptor 2 (HER2) and PI3K pathway which promotes breast cancer progression [163, 164]. In addition, the doxorubicin resistance is caused by the interaction between integrin β1 and galectin 1 [165]; and the cisplatin resistance is also triggered by integrin β1 recruitment and functioning [166]. In lung cancer, integrin β4/PXN/FAK complex mediates cisplatin resistance through regulating ubiquitin specific peptidase 1 (USP1) and voltage-dependent anion channel 1 (VDAC1), which are associated with mitochondrial function and maintaining genomic stability [111]. Integrin β1 also induces erlotinib resistance by regulating the canonical Src signaling. The phenomena indicates that integrin β1 plays an important role in the drug resistance to EGFR-targeted strategy [43].

At present, there are emerging clinic trials focusing on integrin-related nanoparticles for DNA/RNA therapeutics, expected to bring detection and therapeutic promise in the biomedical fields. Integrin α4β7 binding to VCAM-1 and MadCAM-1 induces homing to different tissues. MadCAM-1 is crucial for leukocytes adhesion to intestinal endothelium [167]. A recent study shows that intestinal endothelium generates a recombinant protein containing two domains of MadCAM-1 which has great affinity for integrin α4β7. This study silences interferon γ via lipid nanoparticles targeting the integrin α4β7-MadCAM-1 high affinity conformation and achieves an exciting treatment effect in the experimental colitis [168]. Increasing studies have indicated that nanoparticles carrier RGD peptide efficiently overcome the barriers of DNA transit to target cells. The complex containing high RGD content exerts huge therapeutic effect. Surprisingly, in uterine leiomyoma cells, peptide-based nanoparticles for integrin αvβ3 targeted DNA delivery expand ganciclovir treatment induced cell death [169]. In colorectal carcinoma cells, nanoparticles carrier RGD peptide as well as derivatives of PLGA-tetrac targeting integrin αvβ3 contribute to efficiency of resveratrol treatment on cancer growth and metastasis [170]. These non-viral vehicles-based chemotherapeutic agents combined treatment is of high potential in the clinical translation. Additionally, in PET/CT imaging and photothermal ablation therapy, targeting integrin αvβ3 by copper sulfide nanoparticles carrier RGD peptide effectively improves the side effects associated with the route of administration [171]. Meanwhile, other assembled forms of integrin targeting drugs are also tried in the clinic and preclinical studies, example silica loaded monoclonal antibodies against integrin α2β1 nanoparticles used in macropinocytosis-like mechanism [172], lip ECO-based nanoparticles delivering integrin β3 siRNA used in TNBC [173] and so on.

Natural product compounds targeting integrin and its regulatory components are also proposed as high efficiency, low toxicity and fewer side effects strategy for therapy. Curcumin is a traditional herbal medicine derived from Curcuma longa. Curcumin has been verified as an anti-inflammatory, antiproliferative, antioxidant and antitumor agent used in clinics [174]. In non-small cell lung cancer (NSCLC) and a series of fibrosis-related diseases, curcumin mainly acts on the integrin β1 pathway to suppress proliferation and malignancy [175, 176]. Curcuma also targets integrin α6β4 by regulating AKT/ENPP2 signaling to suppress migration and invasion in breast cancer [177]. Curcumin in combination with resveratrol has been reported to exert an antiangiogenic effect via reducing integrin β3 expression and the inhibitory effect is amplified by the combined form [178]. In addition, resveratrol targeting integrin β1 plays a crucial anti-proliferation and anti-invasion roles in the colorectal cancer microenvironment [179, 180]. An integrin β1/mTOR axis is required for fibroblast differentiation in corneal blindness [181]. Phloretin, a natural product found in apples and strawberries [182], suppresses integrin αvβ3/Src signaling to regulate the actin cytoskeleton during the invasion process of osteosarcoma [183]. Meanwhile, phloretin has been considered as a glucose transporter inhibitor that induces cell death in osteosarcoma cells. It suggests that phloretin treatment enhances the apoptotic sensitivity and cytotoxic effect of chemotherapeutic drugs via mediating integrin αvβ3 and MAPK pathway [184]. Phloretin targeting integrin β3 is also involved in the interaction between leukocyte and endothelial triggered by thrombin during thrombosis and atherosclerosis [185]. Ouabain is the major ingredient of Strophanthus gratus seeds. Ouabain-induced neutrophil migration inhibition is associated with an integrin β2 chain molecule of CD18 and chemokine receptor CXCR2 inflammatory response [186]. Artemisinin, a traditional medicine, is a sesquiterpene lactone compound extracted from Artemisia that acts mainly against malaria. Additionally, artemisinin acts as an antitumor, anti-angiogenic and pro-apoptotic function [187]. Several studies have demonstrated that artemisinin inhibits integrin β3 as well as other receptor-coupled signalings, such as interleukin 1, tumor necrosis factor α, and toll-like receptors in inflammatory and autoimmune diseases, osteoclastogenesis and melanoma [188, 189].

Besides, approximately 260 other drugs targeting various integrin subtypes have already been studied preclinically in academic as well as industry clinical trials [160]. Focusing on integrin in combination with other radiation therapy and chemotherapy treatment strategies may enhance drug sensitivity rather than single-agent treatment. Research on drugs targeting integrin holds promise for treating related diseases.

### Conclusions and future perspectives

Integrin, as a crucial transmembrane receptor, with ‘outside-in’ ligand-binding specificities and ‘inside-in’ signaling properties is not yet fully explored. Abnormal activation of the ‘outside-in’ and the ‘inside-out’ bidirectional signaling machinery of integrin is closely associated with common diseases, such as cancer, chronic inflammation and thrombosis. Although studies on integrin are rapidly expanding, given the number of various integrin subtypes and the complexity of integrin-mediating signals, investigation of how integrin traffics and functions in the development and the course of diseases is still rewarding.

It is already confirmed that the recruitment of talin and probably kindlin are essential for integrin binding to various ligands and activation, which establishes its mechanical sensitivity. Talin and kindlin induce integrin-ligand complex via binding the tail of integrin β, thereby disrupting the α/β ectodomain. Noteworthy, mechanical forces play a similar indispensable role in integrin regulating adhesion-related mechanotransduction. While at this point, talin and kindlin may not cause integrin heterodimer disruption-induced inside signaling but deliver mechanical forces to the integrin-ligand complex. However, whether kindlin plays a similarly crucial role to talin or exerts an extra potential role in integrin stabilization is still unclear and worth to be explored sequentially. When bound to ligands, integrin aggregates and subsequently participates in the actin skeleton network. The integrin-actin axis regulates several intracellular signalings, such as FAK/Src, Rho GTPase, Ras-ERK and Hippo pathway, as well as cellular behaviors, such as proliferation, migration, invasion, apoptosis, survival and cell stemness.

Although numerous studies on integrin have been conducted clinically, however, the approved drugs and therapies are unsatisfactory. Currently, monoclonal antibodies, peptides and small molecules are applied in clinical trials, however, most of the target is on the integrin-ligand binding site or the ligand itself which brings little benefit. Hence, paying more attention to the crosstalk between integrin and other pathways is valuable and will bring unexpected novel opportunities for therapeutics. While the crosstalk between the integrative function of integrins and other pathways, such as TGFβ, Hippo-YAP/TAZ, Wnt-β/catenin and metabolism-related signaling, is initially obscure and complicated as the study progressed, it has become exquisitely clearer. Integrin-dependent regulation of TGFβ is involved in IBD and other immune responses, which could be considered as a therapeutic target. Vedolizumab, as an integrin α4β7 inhibitor, has been used in IBD therapy in clinics. It indicates that integrin-TGFβ signaling will bring increased therapeutic effects. YAP/TAZ nuclear translocation is controlled by integrin β1-Src signaling in skin cancer, basal layer cells as well as other cancer-associated fibroblasts. Dasatinib, as an Src inhibitor, retains YAP/TAZ in the cytoplasm by suppressing integrin β1-Src signaling. Nanoparticles targeting integrin α5 become a novel and effective strategy in metastatic TNBC via modulating Wnt-β/catenin signaling. Besides the synthetic drugs, natural plant compounds as well as nanoparticle-based delivery and RNA-interfered technology have already been used in clinics and enrolled in clinical trials probably due to their high efficiency, low toxicity and fewer side effects.

The tumor microenvironment has been the leitmotif during the research on various cancers. Integrin-mediated mechanosensitivity is unignorable for mechanical cues transmission. In the future, studies on integrin should pay attention to the dependency of integrin and the translational biomarkers to ensure clinical efficacy. To be successful, we should take advantage of genetically engineered models to develop effective diagnostic and therapeutic technologies.

## Data Availability

Not applicable.
